# Awareness and Knowledge of Human Papillomavirus (HPV) Infection and Vaccine Among Women: A Cross-Sectional Study in Jeddah, Saudi Arabia

**DOI:** 10.7759/cureus.45400

**Published:** 2023-09-17

**Authors:** Ashraf Radwan, Hussein Sabban, Rahaf Alsobhi, Nouf Alsayed, Taif Alharthi, Mayar Alzanbaqi

**Affiliations:** 1 Obstetrics and Gynecology, Faculty of Medicine Rabigh Branch, King Abdulaziz University, Jeddah, SAU; 2 Medicine, Faculty of Medicine Rabigh Branch, King Abdulaziz University, Rabigh, SAU; 3 Obstetrics and Gynecology, Faculty of Medicine Rabigh Branch, King Abdulaziz University, Rabigh, SAU

**Keywords:** awareness, infection, women, vaccine, hpv

## Abstract

Background

This study evaluated the comprehension and awareness of human papillomavirus (HPV) infection and vaccination among women in Jeddah, Saudi Arabia.

Methods

A cross-sectional study included 696 female respondents from Jeddah between the ages of 18 and 60. Ethical approval and informed consent were obtained before administering the questionnaire through social media. The data collected included social demographic information and information about HPV and vaccination.

Results

According to the results, only 332 respondents (47.70%) demonstrated good knowledge about HPV. Less than half (47.84%) were aware of the HPV vaccine. However, a slightly higher percentage (53.02%) would be willing to receive the vaccination if it were free. Individuals who were not willing to accept the vaccine believed it had side effects (25.70%), was unsafe (19.92%), or was unnecessary as HPV was a rare infection (17.55%). Demographic variables, such as educational level and occupation, were found to be predictors of HPV knowledge since their corresponding p-values were statistically significant.

Conclusion

It is crucial to raise awareness about HPV and its vaccine among adult women in Jeddah due to the alarmingly low levels of knowledge and awareness. The importance of HPV vaccination must be emphasized repeatedly to ensure that this critical information is understood and acted upon. Overall, there is low knowledge and awareness about HPV and its vaccine among adult women in Jeddah. Therefore, it is imperative to increase awareness while reiterating the importance of HPV vaccination.

## Introduction

Human papillomavirus, or HPV, is considered one of the most common sexually transmitted infections (STIs) worldwide. The infection affects areas such as the vulva, vagina, penis, anus, oropharynx, and the inner surface of the cervix [1]. In addition, the infection has been identified as a cause of many distinct mucosal and cutaneous epithelial lesions and about 15 different cancer types, including oropharyngeal, anal, penile, and cervical cancers. To date, medical professionals have identified over 150 known types of HPV and nearly 40 strains known to infect the genital area. HPV types (6, 11, 42, and 44) are non-oncogenic, whereas other persistent types (such as 16, 18, 31, 33, 35, 45, 51, 52, 58, 59, and 68) are carcinogenic [2]. Anogenital warts and cervical cancer are sexually transmitted diseases caused by HPVs, which are extremely common [3]. Cervical cancer is the second most common form in females between 15 and 44 [3]. Cervical cancer is the sixth leading cause of death for Saudi women between the ages of 15 and 44 and the ninth most prevalent malignancy in the Kingdom of Saudi Arabia (KSA), with one of the lowest incidence rates globally [4]. As a result, the frequency of HPV is low in conservative societies like Saudi Arabia, where sexual relationships are constrained by strict social and religious rules [5]. 

The burden of HPV infections varies worldwide and depends on countries’ geographic location and is modulated by various risk factors such as sexual behaviors, societal norms, and religious inclinations. According to WHO, girls aged 15 years old receive vaccination before they become sexually active, results in the best way of prevention [6]. Also, most Saudi women who have HPV attend hospitals after the infection is already advanced and requires urgent treatments, including radiotherapy, surgery, and chemotherapy [7].

As a result of improved knowledge of HPV infection and vaccination, the number of cervical cancer cases in industrialized nations has decreased dramatically in recent years [8]. According to contemporary research, HPV awareness and understanding are significant determinants of HPV vaccination acceptance. Furthermore, medical practitioners from across the globe have opted to involve parents regarding impacting their decisions on the need to ensure that they are up to date with their children’s HPV vaccination [9]. Also, educating healthcare professionals about various instances of HP infections is fundamental to guarantee that proper protective measures are considered.

Although health practitioners continue to come up with distinguished means of addressing the problem related to HPV infection, it is essential to note that healthcare professionals are more aware of the health concern than the public [10]. Nonetheless, research studies point out that various factors such as higher education, being female, and increasing age have a positive relationship with the issue of HPV awareness. According to different studies highlighting populations from distinct parts of Saudi Arabia, the level or extent of HPV awareness in the country can be somewhat unsatisfactory, as well as related elements such as the HPV vaccine and pap smears [11]. As a result of the lack of awareness on the issue, a significant number of individuals susceptible to the infection may likely be at a greater risk of contracting the virus, which could adversely affect efforts related to reducing the number of HPV infections within the country. Owing to limited research on HPV infections, especially among women in Saudi Arabia, this study hopes to investigate the awareness and knowledge of HPV infection and vaccine among women in Jeddah, KSA.

## Materials and methods

Study design

The study utilized a descriptive cross-sectional design.

Study participants

The focus of this research was on a group of 696 women in Jeddah, Saudi Arabia, who were over the age of 18.

The inclusion and exclusion criteria

The study encompassed solely female residents of Jeddah with an age range between 18 and 60, representing diverse occupations, who consented to be included. It categorically excluded male respondents, females below 18, and those who did not agree to participate.

Study instrument

Between January and March 2023, we distributed a self-constructed Google Form questionnaire to gather information. The questionnaire was based on previous literature and was randomly shared on various social media platforms like WhatsApp, Telegram, and Instagram [12]. We also created an Arabic version to ensure people from diverse backgrounds could access it. The link to the questionnaire can be found at: https://docs.google.com/forms/d/e/1FAIpQLSciq_6Os1QuIyeKtJiSaUl5J4pRxQFqShzX4oPiX7Hn9jE6Pw/viewform?usp=sf_link.

Participants were required to provide informed consent before participating, and the study's primary objective was explained to them. The questionnaire consisted of four sections: the demographic section, awareness items, and questions on vaccination. It is important to note that a knowledge percentage above 75 is considered good, while a ratio between 50 and 75 is only regarded as fair. A knowledge ratio below 50% indicates low knowledge and should be taken care of accordingly.

Data analysis

SPSS version 26 (IBM Corp., Armonk, NY) was utilized to analyze the data set, with both descriptive and inferential statistics being employed. The qualitative data were represented as a percentage, while frequency distribution was utilized to define each variable. Using the Chi-square test, we determined the association between different variables and obtained the corresponding p-value.

Ethical considerations

Ethical approval was secured from the research ethical community at the university, focusing on critical considerations such as informed consent and confidentiality. Respondents were emphatically assured of their right to withdraw consent and abandon the process at any point during the survey.

## Results

The social demographic characteristics of the respondents in terms of their age, marital status, educational level, area of residence, and occupation are presented in Table 1. Regarding age, most respondents (59.63%) fall into the 18-30 age group. The next largest age group is 41-50, making up 17.53% of the respondents, followed by the 31-40 age group at 13.94%. Respondents above 50 comprise the smallest percentage, accounting for 8.91%.

**Table 1:** Social demographic characteristics of the respondents

Variables	Category	Count	Percentages
Age	18-30	415	59.63
	31-40	97	13.94
	41-50	122	17.53
	Above 50	62	8.91
Marital Status	Single	362	52.01
	Married	282	40.52
	Divorced	37	5.32
	Widowed	15	2.16
Educational level	Elementary school	7	1.01
	High school	169	24.28
	Bachelor’s degree	453	65.09
	Higher education	67	9.63
Area of residence	Jeddah	581	83.48
	Outside Jeddah	115	16.52
Occupation	Medical field	168	24.14
	Non-medical field	234	33.62
	Unemployed	294	42.24

Regarding marital status, most respondents (52.01%) are single. Subsequently, 40.52% are married, 5.32% are divorced, and 2.16% are widowed. The highest percentage in terms of the educational level of respondents (65.09%) have a bachelor's degree. This is followed by 24.28% who have a high school education, 9.63% with higher education, and only 1.01% who have completed elementary school. This suggests that the respondents generally have a higher level of education, especially with a significant proportion having completed a bachelor's degree. In terms of area of residence, most respondents (83.48%) live in Jeddah, while the remaining 16.52% reside outside of Jeddah.

Regarding occupation, the most significant respondents (42.24%) are unemployed. This is followed by 33.62% who are in non-medical fields and 24.14% who work in the medical field. This suggests a diverse occupational profile among the respondents, with a significant portion currently unemployed. Overall, the table provides valuable insights into the social demographic characteristics of the respondents. The data highlight the predominant age range, marital status distribution, educational background, area of residence, and occupational profiles of the respondents in the survey. These findings are essential for understanding the sample's demographics and interpreting the study's results in a broader context.

The information regarding the awareness and willingness of respondents to use the HPV vaccination is presented in Table 2. In terms of understanding, 47.84% of respondents indicated that they are aware of the availability of the HPV vaccine, while 52.16% stated that they were unaware of its availability. This suggests a relatively high awareness among the respondents regarding the vaccine. Regarding the willingness to receive the vaccination, 53.02% of respondents expressed their desire, while 46.98% said they would not accept it. This indicates that most respondents are open to receiving the HPV vaccination. The table also explains why respondents would be willing to vaccinate. The highest percentage (27.70%) cited concerns about potential side effects as a reason for their willingness. Other reasons mentioned include perceiving the vaccine as unsafe (19.92%), the fear of injections (8.56%), concerns about rare infections (17.55%), the risk of injury (10.22%), and infectiveness (11.45%). A small percentage (6.6%) gave reasons not covered by the listed categories. These findings demonstrate that while there is a significant level of awareness about the HPV vaccine, some respondents still have reservations or concerns regarding its use. The most common reason for willingness to accept the vaccination is concerns about potential side effects. This suggests that measures to address and provide information about the safety and efficacy of the vaccine would be crucial in increasing acceptance and uptake among the population.

**Table 2:** Awareness about vaccine and willingness to accept it

Awareness and Willingness to use vaccination				
Items	Yes	%	No	%
Do you know that the HPV vaccine is available?	333	47.84	363	52.16
Would be willing to receive the vaccination	369	53.02	327	46.98
Why would you be willing to accept vaccination				
Reasons	%			
Side effects	27.70			
Unsafe	19.92			
Rare infection	17.55			
Fear injection	8.56			
Risk of injury	10.22			
Infective	11.45			
Others	6.6			

Table 3 presents the respondents' knowledge level in crucial items related to HPV. The data show the percentage of respondents who answered “Yes,” “No,” or “I don't know” to each item. In terms of whether HPV is dangerous, 50.29% of respondents answered “Yes,” 5.46% answered “No,” and 44.25% answered “I don't know.” This suggests that most respondents are aware of the potential dangers of HPV. Regarding knowledge of complications related to HPV infection, 33.19% of respondents answered “Yes,” 50.72% answered “No,” and 16.09% answered “I don't know.” Many participants apparently lacked clarity on the complications linked to HPV.

**Table 3:** Knowledge level of the respondents in key items

Items	Yes	%	No	%	I don’t Know	
Do you think that the HPV is dangerous?	350	50.29	38	5.46	308	44.25%
Do you know the Complicatios related to the infection of the HPV?	231	33.19	353	50.72	112	16.09%
Do you think that there is a high possibility of developing the HPV infection?	201	28.88	97	13.94	398	57.18%
Do you know what the types of HPV are?	170	24.43	414	59.48	112	16.09%
Is the HPV infection a rare infection?	110	15.80	376	54.02	210	30.17%
HPV virus is sexually transmitted?	459	65.95	88	12.64	149	21.41%
Cervical Cancer can be the infection of HPV infection?	287	41.24	85	12.21	324	46.55%
Can human papilloma virus cause genital warts?	368	52.87	145	20.83	186	26.72%
Men can develop HPV?	378	54.31	103	14.80	215	30.89%
You can treat HPV with antibiotics?	513	73.71	45	6.47	136	19.54%
Can the injured individuals be without symptoms?	281	40.37	92	13.22	323	46.41%
HPV makes you unable to have children?	134	19.25	387	55.60	175	25.14%
Do you know that the HPV vaccine is available?	177	25.43	481	69.11	38	5.46%

Regarding the possibility of developing HPV infection, 28.88% of respondents believe there is a high possibility, while 13.94% disagree and 57.18% are unsure. There appears to be a problem requiring additional awareness or assurance about the likelihood of developing the infection. Knowledge of the types of HPV presented similarly, with only 24.43% of respondents knowing what the classes are. 59.48% answered “No,” and 16.09% answered “I don't know.” This indicates a lack of knowledge about the different types of HPV.

Regarding the transmission of HPV, 65.95% of respondents correctly identified that it is sexually transmitted, while 12.64% answered “No,” and 21.41% answered “I don't know.” The data show that the participants understood the transmission of HPV adequately. In terms of cervical cancer, this HPV, and other factors can cause cervical cancer. HPV and other factors can cause cervical cancer. infection, 41.24% of respondents answered “Yes,” 12.21% answered “No,” and 46.55% chose “I don't know.” HPV and other factors can cause cervical cancer. Based on the responses, some participants seemed uncertain about the link between HPV infection and cervical cancer. Knowledge about whether HPV can cause genital warts is relatively good, with 52.87% of respondents answering “Yes,” 20.83% answering “No,” and 26.72% saying “I don't know.” This suggests that the respondents reasonably understood the association between HPV and genital warts. 

Regarding whether men can develop HPV, 54.31% of respondents correctly answered “Yes,” 14.80% answered “No,” and 30.89% chose “I don't know.” You appear to have a commendable understanding of HPV and its potential effects on both genders. Lastly, when asked if HPV can be treated with antibiotics, 73.71% of respondents answered “Yes,” 6.47% answered “No,” and 19.54% answered “I don't know.” This suggests a widespread misconception among respondents as HPV cannot be treated with antibiotics. The table presents a concerning blend of comprehension and misunderstandings regarding significant facets of HPV among the participants. Although awareness about some aspects, like the hazards and transmission of HPV, is moderately adequate, there is an urgent requirement for more education and information in other domains, such as the specific strains associated with HPV infection and the misbelief that antibiotics can cure HPV.

The association between social demographic variables and awareness of the availability of the HPV vaccine is presented in Table 4. Age: the data show no significant association between age and understanding of the vaccine. The awareness percentages are relatively similar across different age groups, with the highest rate observed among those aged 18-30 (50.12%). Marital status: based on the available data, no evident correlation exists between one's marital status and vaccine awareness. Educational level: there is a significant association between academic level and vaccine awareness. Individuals with higher levels of education, such as a bachelor's degree or higher education, have a higher percentage of understanding (55.41% and 59.70%, respectively) compared to those with a lower educational level, such as elementary school or high school. Area of residence: the data do not indicate a significant association between the place of residence (Jeddah or outside Jeddah) and vaccine awareness. Occupation: there is a significant association between occupation and vaccine awareness. Individuals working in the medical field have a significantly higher percentage of understanding (85.12%) than those in non-medical areas or unemployed.

**Table 4:** Association between social demographic variables and awareness

Variables	Category	Aware	%	Not Aware	%	P-value
Age	18-30	208	50.12	207	49.88	
	31-40	46	47.42	51	52.58	0.456
	41-50	58	47.54	64	52.46	
	Above 50	20	32.26	42	67.74	
Marital Status	Single	134	37.02	228	62.98	
	Married	174	61.70	108	38.30	0.345
	Divorced	16	43.24	21	56.76	
	Widowed	8	53.33	7	46.67	
Educational level	Elementary school	2	28.57	5	71.43	
	High school	39	23.08	130	76.92	0.011
	Bachelor’s degree	251	55.41	202	44.59	
	Higher education	40	59.70	27	40.30	
Area of residence	Jeddah	290	49.91	291	50.09	0.567
	Outside Jeddah	42	36.52	73	63.48	
Occupation	Medical field	143	85.12	25	14.88	
	Non-medical field	112	47.86	122	52.14	0.042
	Unemployed	77	26.19	217	73.81	

In summary, the data suggest that educational level and occupation are the social demographic variables most strongly associated with awareness of the HPV vaccine. Higher education and medical fieldwork are associated with more heightened vaccine awareness. These findings can inform targeted awareness and education campaigns towards specific demographic groups with lower levels of understanding.

The data on the willingness to accept the HPV vaccine and its association with the level of knowledge about the vaccine is seen in Table 5. The data show that individuals with good knowledge about the vaccine are significantly more willing to accept it than those with poor knowledge. Among those with good knowledge, 72.29% expressed willingness to get the vaccine, while only 24.7% stated they would not accept it. On the other hand, among those with poor knowledge, a lower percentage (35.44%) expressed their willingness to get the vaccine, while a higher rate (64.56%) stated that they would not be willing to accept it. This association between knowledge and willingness to accept the vaccine is statistically significant, as indicated by the p-value of 0.001. These findings underscore the importance of knowledge and information in shaping individuals' attitudes and decisions regarding the HPV vaccine. It suggests that efforts to improve knowledge and awareness about the vaccine may contribute to increasing the acceptance and uptake of the vaccine among the population.

**Table 5:** Willingness to accept vaccine and the association with the level of knowledge.

		Willingness to accept vaccine				Total	P-value
Awareness		Yes	%	No	%		
	Good knowledge	240	72.29%	82	24.70%	332	0.001
	Poor knowledge	129	35.44%	235	64.56%	364	

## Discussion

This research assessed the comprehension and familiarity of HPV infection and vaccines among adult females residing in Jeddah. Most respondents (83.48%) live in Jeddah, while the remaining 16.52% reside outside of Jeddah, and those living in rural areas outside Jeddah responded poorly. This indicates that the survey sample primarily encompasses individuals living in Jeddah, the outcomes suggested that the knowledge regarding HPV was relatively low, with only 47.70% of participants having adequate understanding. These findings align with a previous study conducted in the western region of KSA, which reported an awareness level of 34.6% [13]. Different demographic factors can influence the amount of HPV knowledge. It is essential to take note of the findings in this study, as they have shown a significant and undeniable link between education level and expertise. Our study showed a statistically significant association between awareness related to HPV infection and HPV vaccines with the education level and occupation.

With the rising incidence of cervical cancer and the risks associated with HPV, vaccination against HPV is deemed imperative. The WHO recommends vaccination of HPV for both sexes at the age of 9-14 years, followed by another booster vaccination for females between the ages of 13 and 26 years. This study's questions about vaccine awareness and willingness to get vaccinated were crucial. Upon questioning the vaccine, it appeared that just 47.84% of the participants were acquainted with its existence. However, more than half of the respondents (53.02%) would be willing to receive the vaccine. Some respondents who were against the vaccine asserted that they felt it had side effects (25.70%), was not safe (19.92%), or was practical (11.45%), among other reasons. The study by Alkhaldi et al. [13] found that nearly 75% of the respondents would be willing to get vaccinated, and the majority who were unwilling to get vaccinated felt that it was unnecessary (32.5%).

We investigated if being aware of HPV was linked to the willingness to receive the HPV vaccine. The p-value obtained was statistically significant (p-value = 0.001). Furthermore, those who knew HPV were more inclined to get vaccinated (72.29%). The results of this study align with a previous one conducted among Indian women, where over 88% of those who knew about HPV expressed their willingness to take the vaccine [14]. It is worth noting that a lack of knowledge about HPV and vaccination is widespread. Furthermore, occupation appears to play a role in HPV awareness, with individuals in the medical field demonstrating a notably higher level of understanding than those in other professions [15].

Furthermore, research has indicated a correlation between occupation and knowledge. A study in Riyadh has suggested that age and education levels predict awareness, whereas employment status does not appear to be a significant predictor [15]. It is essential to take these results seriously when making decisions in related fields.

It is important to note that this study has some limitations. The population sample in this study does not represent the general population in Jeddah. Furthermore, individuals in the medical field are generally more knowledgeable than the general public regarding infections and vaccines. The data collected was self-reported by women, which means that factors like recall or social desirability bias could have influenced the results. It means that some participants may have either over or under-reported their awareness and knowledge about HPV infection and the vaccine, which could potentially skew the study's findings.

Additionally, the limited access to healthcare among women may have affected the study's findings. The study's findings are significantly constrained since data were collected through self-reports, which were only obtainable to those with access to healthcare facilities. Therefore, the knowledge levels observed in the study may only partially reflect the knowledge levels of the entire female population. It is essential to consider the limitations of this study. The data were provided by women, which may have been influenced by factors such as memory recall bias or an inclination to present oneself positively. This means that some participants may have over or under-reported their knowledge and awareness of HPV infection and the vaccine, potentially impacting the accuracy of the study's results.

Considering the cultural norms and beliefs prevalent in Jeddah is imperative when analyzing women's responses regarding HPV infection and vaccines. It is essential to assess the impact of these factors, as they could have had a significant effect on the accuracy and validity of the study.

## Conclusions

The study aimed to evaluate the knowledge level about HPV and its vaccination among adult women in Jeddah, Saudi Arabia. The results showed that most women in Jeddah are unaware of HPV and its vaccination. The study also revealed that there is a correlation between the level of education and awareness of HPV. Additionally, there was a significant association between occupation and awareness. The study found that women knowledgeable about HPV were more likely to accept vaccination. It is crucial to increase awareness about HPV and emphasize the importance of vaccination to promote better health outcomes.
